# Understanding barriers preventing pregnant women from starting antenatal clinic in the first trimester of pregnancy in Ntcheu District-Malawi

**DOI:** 10.1186/s12978-018-0605-5

**Published:** 2018-09-21

**Authors:** Chancy S. Chimatiro, Precious Hajison, Effie Chipeta, Adamson S. Muula

**Affiliations:** 1 Department of Health Systems and Policy, School of Public Health and Family Medicine, College of Medicine, University of Malawi, Blantyre, Malawi; 2 PreLuHa consult, Namiwawa Street, Newroard location, PO BOX 703, Zomba, Malawi; 3 Department of Public Health, School of Public Health and Family Medicine, College of Medicine, University of Malawi, Blantyre, Malawi; 4 Centre for Reproductive Health (CRH), University of Malawi, College of Medicine, Blantyre, Malawi; 5 Africa Center of Excellence in Public Health and Herbal Medicine(ACEPHEM), University of Malawi, College of Medicine, Blantyre, Malawi

**Keywords:** Antenatal attendance, First trimester, Focused antenatal care, Pregnancy

## Abstract

**Background:**

Exploring barriers contributing to low utilization of Antenatal Care (ANC) during the first trimester of pregnancy is of national programmatic importance. We conducted an exploratory study in 2013 at Bilira Health Centre in Ntcheu district-Malawi with an aim of understanding barriers that prevent pregnant women from attending antenatal clinics in the first trimester of pregnancy.

**Method:**

This was cross sectional exploratory study using qualitative approach. Data were collected from ANC clients, key informants, health services professionals and women of child bearing age (15–49 years) using an in-depth interviews and Focus Group Discussions (FGDs). Data were analysed manually by reading the transcriptions and memos several times inorder to be familiar with the themes emerged. The emerged themes were coded.

**Results:**

Most of the women reported that they have a feeling of starting ANC in the early days of their pregnancies, however, they also reported several barriers ranging from cultural beliefs, social economic to service delivery barriers. On cultural barriers many women wait for marriage counselors from husband’s side to give them advice before starting ANC in the process called “*Kuthimba*”. Some women hide the pregnancy in early months to avoid being bewitched. On social-economic barriers, some of the women mentioned that they don’t start ANC early waiting for new clothes. Poor attitude of health workers also has an effect on ANC attendants. Most women pointed out that they started ANC late because some health workers were rude and do not observe confidentiality. Men’s refusal to accompany their spouses to antenatal clinic in fear of HIV test and some by-laws which restrict women who had pregnancy outside marriage to seek an authorisation letter first from Traditional Leaders for them to start ANC at the health facility were also mentioned as contributing barriers.

**Conclusion:**

Women should be oriented on the national guidelines on Focused ANC (FANC) which advocates for at least 4 visits. There should also be Information, Education and Communication (IEC) on ANC and interventions to deal with social-cultural issues while at the same time improving service delivery at the health facility so that ANC services can be accessible and responsive enough.

## Plain English summary

Antenatal attendance during the first trimester is still low in most of the developing countries especially Sub-Saharan region including Malawi. First trimester of pregnancy is defined as a period within the first 3 months of pregnancy. This study was conducted inorder to find out what barriers prevent women from attending antenatal clinics (ANC) in the first trimester of pregnancy and what should be done to improve the situation.

Participants were asked to mention what they think prevent pregnant women from starting ANC in the first trimester. Key Informants Interviews (KIIs) and Focus Group Discussions (FGDs) were conducted.

In all KIIs and FGDs most of the women had positive perception on starting ANC early, however, they also reported several barriers ranging from cultural beliefs, social economic to service delivery barriers.

In conclusion, inadequate knowledge on the importance of attending ANC in the first 3 months of pregnancy was main contributing factors in most of the women. Therefore, this study recommends that Information, Education and Communication (IEC) on ANC should be prioritized among women if the trends is to be reversed.

## Background

Antenatal care is the care provided to a woman from the beginning of pregnancy until the onset of labour [1]. This care often involves recording history, assessment of individual needs, advice and guidance on pregnancy and delivery, screening tests, education on self-help care during pregnancy, identification of conditions detrimental to health during pregnancy, first line management and referral if necessary [2]. Malawi’s current guidelines are derived from the WHO/UNICEF report 2003, which recommended that women attend formal antenatal care services for at least 4 times during each pregnancy [3]. The visits are divided into trimesters namely first trimester, second trimester and third trimester. Ideally the first visit should be within 12 weeks of pregnancy but not later than 16th week of pregnancy [4]. The second visit should be within 24 to 28 weeks, the third visit shouldc be at 32 weeks and forth visit at 36 weeks of pregnancy.

Early initiation of antenatal care can be beneficial through: early identification of risk factors and provision of preventive and health promotion advice to encourage healthy lifestyles, treatment of medical conditions such as diabetes and pregnancy induced hypertension and referrals to services such as nutrition support and smoking cessation programs [5]. Some studies have reported that women who have few or inadequate visits and those who start antenatal care late than the first trimester have poorer pregnancy outcomes, such as low birth weight and pre-term birth [6, 7]. However, increasing the number of antenatal visits does not necessarily improve the outcomes of pregnancy [8] but should be responded with quality of care.

Globally about 70% of women attend antenatal visit at least once during pregnancy while in high income countries 98% of women attend antenatal visit at least once [9]. In low and middle-income countries and sub-Saharan Africa region ANC attendants is still low. Low et al. reported that only 65% and 68% of women report for at least one antenatal visit during pregnancy in low and middle-income countries and sub-Saharan region respectively [10].

Antenatal attendance during the first trimester shows good to poor coverage in different regions of the world. For example, about five out of six mothers (84%) begin antenatal care in the first trimester in the United States (US) [8]; 70% in Latin America/ Caribbean; about 68% in Middle East / North Africa region, 50% in Asia and lowest attendance in the first trimester is in Sub-Saharan region 24% [11]. Some countries have particularly low attendance; a study in Nigeria showed that only 22.8% of the women who received ANC booked in the first trimester [12]. In Kwa-Zulu- Natal, South Africa, most women (860/872; 98.6%) attended the antenatal clinic during their pregnancy of which only 72/860 (8.4%) women reported starting ANC in the first 3 months of pregnancy [13].

In Malawi, 12% of women had ANC visit in the first trimester; about 48% had their first antenatal visit between 4 and 5 months of pregnancy while 36% started at 6 and 7 months of their pregnancy [4]. And 2015–16 Malawi Demographics and Health Survey (MDHS) revealed that only 24% of women had their first ANC visit during the first trimester, 51% of women had their first ANC visit during the fourth and fifth month of pregnancy, and 2% did not receive any ANC until the eighth month or later [14]. Ideally, all pregnant women are supposed to have their first antenatal visit during the first trimester [15]. According to MDHS, 2010, there was no significant difference between urban and rural pregnant women in antenatal attendance in Malawi [4].

A study conducted to investigate factors that contribute to low antenatal attendance during the first trimester among the Sena of the lower shire in Nsanje, Malawi found that there was inadequate knowledge about the ideal time of starting ANC. Antenatal care clinics were understaffed and traditional beliefs and cultural practices were reported to affect timing for antenatal care [16]. Mgawadere F.M (Mgawadere FM: Assessing the quality of antenatal care at Lungwena health Centre in rural Malawi, unpublished) in a study assessing quality of antenatal care at Lungwena in Mangochi district, Malawi, found that pregnant women started ANC late in second and third trimester. Non-attendance to ANC during the first trimester was associated with social cultural practices such as afraid of witch craft, long distances and need for material support from family members [16]. Therefore, this current study aimed at exploring barriers preventing pregnant women from starting ANC in the first trimester of pregnancy in Ntcheu district-Malawi. Specifically, we explored perception of pregnant women towards ANC, social cultural and health service barriers that affect ANC attendance in the first trimester of pregnancy.

## Methods

This was an exploratory cross sectional study using a qualitative approach. Data collection was done in 2013.

### Study setting

This study was conducted in the community of Bilira Rural Health Centre in Ntcheu district. In 2010/2011 Ntcheu district registered 46,786 women who attended antenatal clinic and out of these 3462 women attended the antenatal clinic during the first trimester which represents 7.4% [17]. Bilira Health Centre had a total catchment population of 45,187 and with 58 villages. It had 11,363 women who were of reproductive age group (15–49 years).

### Sampling technique

Convenient sampling was used to identify participants for in-depth interviews at the health centre. Purposive sampling technique was used to select participants for FGDs and key informants. We randomly selected twelve villages where FGDs were conducted in each village. Key informants included were health workers, traditional leaders, traditional birth attendants and religious leaders.

There were 10 pregnant women, 3 antenatal health services workers and 9 key informants that took part in depth interviews. A total of twelve FGDs categorized into 3 different age groups were conducted. The categories were as follows; four groups of women of ages between 15 and 24 years, another four groups of 25–34 years and last four groups of 35–44 years of age. Each FGD had minimum of eight participants and maximum of twelve participants. In total there were 126 participants in FGDs. The maximum study participants was reached at by looking at saturation of responses.

### Data collection

In-depth interviews were conducted using open ended questions. The main thematic areas explored were on knowledge and motivations about ANC attendance, perception towards starting ANC early, Social-cultural practices, Social-economic factors and factors associated with access to ANC services. In both interviews and FGDs, digital recorders were used to collect data in the local language (Chichewa).

### Data management

The proceedings of the interviews were listened to several times before transcribing the data in Chichewa which was later translated in English. Data was analysed manually by reading the transcriptions several times inorder to be familiar with it and explore the themes emerged from transcriptions and memos. The emerged themes were given appropriate codes. The codes were being assigned under the main theme it supported.

The main ideas from each participant concerning ANC were isolated and assigned under the theme in which it supported. The ideas were analysed to come up with final findings of the study.

### Ethical considerations

The study protocol was reviewed and approved by the College of Medicine Research and Ethics Committee (COMREC) to ensure that ethical issues had been addressed under research approval No SP1212102. Permissions to carry out the study were obtained from District Health Office (DHO) and the District Commissioner (DC) for Ntcheu and Traditional Leaders around Bilira Health Centre.

## Results

A total of 22 in-depth interviews and twelve FGDs were conducted. We grouped the results in the following themes; knowledge on ANC attendance, perception towards early ANC attendance, social-cultural practices, social-economic factors and factors related to access of service delivery.

### Knowledge and motivations on starting ANC early

Through in depth interviews with ANC clients, Key Informants and FGDs, we found that most women had knowledge on time of starting ANC. Their understanding was that it is important to start ANC during the first trimester. We also found that most women understood benefits of starting ANC early such as getting early pregnant care and getting prompt treatment if someone has low blood level. Most women also understood the disadvantages of starting ANC late such as not knowing the position of the baby and also not knowing what kind of supplementary food to take.

A number of women who participated in the in-depth interviews demonstrated knowledge of starting ANC in their first trimesters but instead reported to deliberately started ANC in either second trimesters or third trimester. Two of the women said, *“I started when I was 5 months pregnant so that I can reduce number of visits of attending ANC before I deliver,*” (ID No 6) (ID NO 10).

### Perception towards early ANC attendance

There was overall positive perception among women towards starting ANC early. The study found that most of the women were not starting ANC early because their partners refused to accompany them as they were afraid of being tested for HIV. Some woman reported in separate FGDs, saying “*men were the main barriers to starting ANC early in fear of HIV test*” (FGD No 2, Client ID No 8) (ID No 5, RESPO No 6).

### Social-cultural practices

#### Authorisation from husband’s side

In both FGDs and in depth interviews, participants were asked about cultural practices in the area. We found that many women wait for marriage counselors from husband’s side to come and give them advice before starting ANC in the process called “**Kuthimba**”. A woman said, *“Some of us don’t start ANC early as we are supposed to wait for marriage counselor from man’s side to come and give us some advises”* (ID No 6, FGD 4). Another woman said*, “it is our usual practice here to inform elders from man’s side to come and give advise to the woman especially when it is her first pregnancy”* (ID No 9, key informant).

#### Beliefs regarding witch craft

We found that hiding the pregnancy in early months to avoid being witched was contributing to low ANC attendance during the first trimester. A Key informant said, *“I think most women here don’t start ANC early because they normally try to hide the pregnancy in its early stages to avoid being witched”* (ID No 5). Another woman said during FGDs 6, “*elderly women give some of us traditional medicine that protects the pregnancy from being be witched and advise you to wait for the pregnancy to grow before starting ANC”* (ID No 3).

### Social-economic barriers

#### Economic status

We found that social-economic factors have an effect on the time for starting ANC as many of women don’t start ANC early waiting for new clothes. This is so due to poor socio-economic status (poverty) of most families in the area. Many women do not start ANC earlywaiting for their husbands to buy them new clothes in order to attend ANCin newly and nice looking clothes. A woman said, *“When one is pregnant she normally waits for the husband to buy new clothes so that she can start ANC in new and nice looking clothes”* (ID NO 8, in depth interviews).

The opinions of the FGD participants and KIIs were very similar on the dilemma that arises between the need to provide for the family versus the need to start ANC. This study showed that business had an effect on decision to start ANC early. Some women preferred going for business rather than starting ANC when they had less than 5 months pregnancy inorder to provide for their families. This signifies that social-economic is indeed another factor affecting decision to start ANC early. Another respondent said, *“If you have millet and it is a market day while you are also supposed to go for ANC, it is better to go to the market and sell millet so that you can have something to feed your family”* (ID No3, FGG 2).

We found that farming has little effect on starting ANC early. This is so as most of the women said that farming cannot prevent someone from starting ANC early because there are several months in the year for farming and ANC schedule is once a month. A woman said, *“I cannot fail to start ANC early because of farming as ANC schedule is only once a months while farming is our daily work for several months”* (ID NO 7, in-depth interviews).

### Access to services

Long distances from home to the facility have effects on the decision to start ANC early. Some women reported to travel more than 7 km (often by foot) in order to access ANC services. This prevents women for starting ANC early in the first trimester. A woman said, *“I cannot start ANC early because I come from very far from the clinic, I do not want to have many visits and get tired before delivery”* (ID NO 7, in depth interviews). Another participant had this to say, *“When I think of distance from home to here I always get discouraged to start off”* (ID NO 6, in depth interviews at the facility).

### Male involvement

We found that many women do no start ANC early if their husbands are away or refuse to accompany them. Women may also not initiate ANC if they do not have a spouse. In this study area, there was a by-law which advocates that every woman should attend ANC clinic with her spouse during the first visit. This was being reinforced by traditional leaders around the area. If women go to the health facility without their partners they are not attended to as a result most women do not start ANC early. We also found that if the woman has no partners, she is supposed to get an authorisation letter from the traditional leader in order to access ANC services at the health facility otherwise, she cannot be attended to.

A woman said during one of the FGDs, *“if my husband has refused to accompany me, then I cannot go to start ANC alone because I know that I will not be attended to”* (ID No 3).

Another woman said, “*I started ANC when I was five months pregnant as I was waiting for my husband who was away by then”* (ID No 1).

Another woman said, *“if you are pregnant outside marriage then you are supposed to go and get an authorisation letter from village headman for you to be allowed to start ANC”* (FGD 5, RESPO NO 7).

### Attitude of health workers

During both FGDs and in depth interviews we found that poor attitude of health workers towards clients has an effect on decision to start ANC early. We found that some health workers at the facility rarely observed confidentiality. A woman said during one of the FGDs, *“there is a certain nurse there who even reveals what you have worn inside your wrapper (Chitenje)”* (ID No 6, FGD 5).

Another Key informant said, “*I have been receiving complaints from women concerning the way they are being handled at the clinic, in fact some of the providers are rude to our women”* (ID No 9).

### Waiting time

Study participants reported that, there was long waiting time for the ANC women to access services at the facility due to integration of the service with family planning. This claim was confirmed by the study team during the key informant interviews with pregnant women at the facility as they were waiting for the ANC clinic to start.

It was reported that health workers were prioritizing family planning clients before starting attending to ANC clients. A woman said during an in-depth interview, *“I always come for ANC early so that I can be assisted and go back on time, however, nurses give priority to those who have come for family planning first than us, as a result we usually go back home very late”* (ID No 9 for ANC Clients).

## Discussion

Having adequate knowledge about optimal time for starting ANC, its benefits and the downside associated with starting ANC late may not assure that women can start ANC early. Most women reported to have started ANC late with an aim of reducing number of visits before delivery. Similarly, another study done in Nsanje reported that some women started ANC late because they wanted to reduce number of visits so that they should not get tired before delivering [16].

Cultural practices have a role in determining time for starting ANC. We established that some women do not start ANC early as they wait for marriage counselors or their mother in-laws to come and give them advice especially if it is the first pregnancy. This leads to the pregnancy being kept secret until it reaches 3 to 4 months old. These findings have been noted elsewhere. Studies done in Nsanje and Nepal reported that women wait for their mother in-laws to either give them advise or make decision for them to start ANC [16, 18].

The belief that witchcraft could impact (early) pregnancy was reported to prevent women form starting ANC early as most women were influenced to hide the pregnancy and also took traditional medicine to protect the pregnancy in its early stages. These findings support what was reported in another study in Nsanje that most women were afraid to be bewitched if people discovered that they were pregnancy especially during early stages of pregnancy [16].

Socio-economic factors also assist in the decision to start ANC early. We found that some women do not start ANC early because they demand new clothes from their husbands. This was also reported in other studies done in South Africa and Nigeria where women were reported to start ANC late because they needed to find money for transport and new clothes [19, 20]. The finding of the studies shows that socio-economic status may be associated with the decision to seek health services especially on time to start ANC.

Some women are involved in small scale businesses in order to feed their families. Our study noticed that there was competing interest in decision making whether to go for business or ANC. This activity affects the decision to start ANC early as most women prefer going for business so that they can get money to buy new clothes for ANC. Similarly this was also reported in another study done in Blantyre, Malawi, which reported that some men failed to participate in accompanying their wives because of work or business obligations [21].

In addition, we report that long waiting time has also negative impact in decision to start ANC early. In another study done in Benin, they reported that waiting time was one of the determinants of low utilization of ANC during the first trimester [22].

Long distances from health facilities limit access to health care when required. This study agrees with other studies done elsewhere which reported about long distances from homes to the health facility as a barrier to access medical care [23–25].

Male involvement plays important role in encouraging women to start ANC early as some women do not start ANC early because their husbands refuses to accompany them. This has been also reported in other studies [19, 26]. Poor attitude of the health workers prevents some women from starting ANC early. Disrespectful care has been reported in different settings and impact negatively on the quality of services [21, 27, 28]. This is the strongest predictor about how responsive service delivery is to those who need them as reported by another study done Sweden about women’s expectations on ANC services [29].

## Conclusion

Although women may have positive perceptions towards starting ANC early, they are prevented from doing so due to various barriers. These barriers range from socio-cultural factors to service delivery factors.

We recommend that public facilities should put clients at the centre of health services by providing good quality care with respect. Therefore, a well-functioning health work-force is the one that is responsive to the needs of its users or customers. As such health systems managers should train health workers on customer care so that they can change their attitude towards women who attend ANC. This will make pregnant women not to be scared to go for ANC.

We also recommend that traditional leaders, being custodians of custom, should come up with deliberate by-laws which can help in addressing some social-cultural practices so that women should start ANC early. Policy makers should ensure that there is enough IEC materials to sensitize women on the need to start ANC early. Women empowerment through education should be enhanced so that they can make independent and reasonable decisions concerning their health and that of their unborn baby. Availability of mobile clinics with integrated services, which include ANC, should be encouraged at all level especially in hard to reach areas inorder to reduce distance covered by pregnant women to access ANC services from health facility.

## Abbreviations

ACEPHEM: Africa Centre of Excellence in Public Health and Herbal Medicine
ANC: Antenatal Care
COMREC: College of Medicine Research and Ethics Committee
CRH: Centre for Reproductive Health
DC: District Commissioner
DHO: District Health Officer
FANC: Focused Antenatal Care
FGDs: Focus Group Discussions
ID: Identification No
IEC: Information, Education and Communication
KIIs: Key Informant Interviews
MDHS: Malawi Demographics and Health Survey
RESPO: Respondent
UNICEF: United Nations International Children Education Fund
WHO: World Health Organisation
